# Autoimmune-Mediated Thymic Atrophy Is Accelerated but Reversible in RelB-Deficient Mice

**DOI:** 10.3389/fimmu.2018.01092

**Published:** 2018-05-22

**Authors:** Brendan J. O’Sullivan, Suman Yekollu, Roland Ruscher, Ahmed M. Mehdi, Muralidhara Rao Maradana, Ann P. Chidgey, Ranjeny Thomas

**Affiliations:** ^1^Diamantina Institute, Translational Research Institute, University of Queensland, Princess Alexandra Hospital, Brisbane, QLD, Australia; ^2^Stem Cells and Immune Regeneration Laboratory, Department of Anatomy and Developmental Biology, Monash University, Clayton, VIC, Australia

**Keywords:** thymic atrophy, RelB, autoimmune disease, polymorphonuclear cells, immunotherapy

## Abstract

Polymorphisms impacting thymic function may decrease peripheral tolerance and hasten autoimmune disease. The NF-κB transcription factor subunit, RelB, is essential for the development and differentiation of medullary thymic epithelial cells (mTECs): RelB-deficient mice have reduced thymic cellularity and markedly fewer mTECs, lacking AIRE. The precise mechanism of this mTEC reduction in the absence of RelB is unclear. To address this, we studied mTECs and dendritic cells (DCs), which critically regulate negative selection, and thymic regulatory T-cells (tTreg) in RelB^−/−^ mice, which have spontaneous multiorgan autoimmune disease. RelB^−/−^ thymi were organized, with medullary structures containing AIRE^−^ mTECs, DCs, and CD4^+^ thymocytes, but fewer tTreg. Granulocytes infiltrated the RelB^−/−^ thymic cortex, capsule, and medulla, producing inflammatory thymic medullary atrophy, which could be treated by granulocyte depletion or RelB^+^ DC immunotherapy, with concomitant recovery of mTEC and tTreg numbers. These data indicate that central tolerance defects may be accelerated by autoimmune thymic inflammation where impaired RelB signaling impairs the medullary niche, and may be reversible by therapies enhancing peripheral Treg or suppressing inflammation.

## Introduction

Medullary thymic epithelial cells (mTECs) develop from immature mTECs that express the lectin UEA-1 and low levels of MHC and costimulatory molecules. Extrinsic signals such as RANKL, CD40L, and lymphotoxin-beta promote differentiation of mTECs to mature cells with increased MHC, CD80 and AIRE expression (1, 2). The NF-κB transcription factor subunit, RelB, and NF-κB-inducing kinase (NIK) upstream of the non-canonical NF-κB pathway, are essential for the development and differentiation of mTECs (3). RelB-deficient mice have reduced thymic cellularity and markedly fewer mTECs (4). However, RelB-deficient mice have reduced lifespan due to multiorgan autoimmune disease and severe thymic medullary atrophy which limits interpretation of effects of RelB on mTEC differentiation or function (5). Thus, the precise role played by RelB in mTEC differentiation is unclear.

Furthermore, while mTECs have been shown to support the development of thymic (t)Treg in the thymus, the role of RelB in this process is unknown. MHC and CD40/CD154 interactions are required together with CD80/86 co-stimulation, thus implicating mature mTECs in thymic regulatory T-cells (tTreg) development (6, 7). The role of RelB is particularly relevant, as RelB^−/−^ thymi were shown to lack UEA-1, which is a pan-marker of mTECs expressed after their differentiation from bipotent TEC progenitors (4). When Treg development was studied in the context of RelB deficiency, fewer tTreg and fewer Foxp3^−^CD25^+^CD69^+^CCR7^+^CCR9^−^ tTreg precursors developed in wild type mice engrafted with RelB^−/−^ than wild type thymi (8). However, RelB^−/−^ thymic grafts not only had a marked reduction in ERTR5^+^ mTECs but also demonstrated a severely restricted medullary area.

Despite this reduction in tTreg precursor development in RelB^−/−^ thymi, peripheral Treg (pTreg) are present at increased frequency in the spleen and disease-affected tissues of RelB^−/−^ mice. RelB^−/−^ Treg are functionally defective due to deficient dendritic cell (DC) expression of MHC class II and costimulatory molecules required for effective TCR signaling (9). Adoptively transferred RelB^+^ DCs are able to license pTreg to suppress autoreactive T cells and inflammatory disease, demonstrating that pTreg developing in RelB^−/−^ mice are functional when appropriately signaled. Furthermore, functional pTreg are essential for dominant tolerance in this model (9). Although these results demonstrate that pTreg develop through RelB-independent mechanisms, the relationship between tTreg development and RelB-deficient thymic antigen-presenting cells (APCs) remains unclear.

Medullary dendritic cells (DCs) interwoven with mTECs also contribute to tTreg development (10). mTECs can provide signals to DCs such as XCL1, which localizes DCs to the medulla. XCL1-deficient mice have reduced tTregs (11). In addition, inhibition of Delta-like ligand-4/Notch signaling enables DN1 T cell progenitors to differentiate into DCs to expand tTregs, and mTECs expressing NIK are required for expression of costimulatory molecules by thymic DCs (3, 12). These results indicate that expansion of mature DCs within the thymus correlates with increased tTreg development. However whether this is directly due to DCs or results from mTECs promoting tTreg development and recruiting DCs to the thymus is currently unknown. Furthermore, the relative impact on tTreg development of mTEC and DC RelB expression has not been determined. In turn, extrinsic signals from activated T cells are required to activate RelB expression for the development of mature mTECs. Therefore, we studied the impact of RelB deficiency on medullary development, mTECs, DCs, and tTreg cell generation in the thymus.

## Materials and Methods

### Mice

All mice were maintained in specific pathogen-free conditions. Mice homozygous for an insertional mutation in the RelB gene (designated RelB^−/−^), on a C57BL/6 background, were generated as described (5) and used for experiments at 4–9 weeks of age. RelB^+/−^ mice displaying neither pathology nor immune dysfunction, or C57BL/6 (RelB^+/+^) were used as controls. RelB^−/−^ radiation chimeras [bone marrow chimeras (BMC)] were generated by transferring bone marrow from RelB^−/−^ or RelB^+/−^ into lethally irradiated C57BL/6 hosts (Animal Resource Centre, Perth, WA, Australia). Mice were used after at least 8 weeks of reconstitution. All experiments were approved by the University of Queensland Animal Ethics committee. All mice were housed in a pathogen-free environment.

### Treatment of RelB^−/−^ Mice

CD11c^+^ DCs were isolated from disaggregated, collagenase-treated RelB^+/−^ spleens using CD11c-conjugated immunomagnetic beads (Miltenyi Biotec, Bergisch Gladbach, Germany). Five million splenic RelB^+/−^CD11c^+^ DCs were injected into RelB^−/−^ recipients intravenously. For granulocyte depletion, RelB^−/−^ mice were administered 500 µg of anti-Ly6G (clone 1A8, BioXcell) antibody twice weekly for 4 weeks. For expansion of thymic DC, RelB^−/−^ or RelB^+/+^ mice were administered Progenipoeitin-1 (Pharmacia, St. Louis, MO, USA), subcutaneously 20 μg/animal per day for 10 days.

### Analysis of mTECs

Individual collagenase-treated, disaggregated thymi were stained for MHC class II, Ly51, and CD45.1 (A20) and analyzed by flow cytometry. mTECs were CD45.1^−^Ly51^−^MHCII^+^, and cTECs were CD45.1^−^Ly51^+^MHCII^+^.

### Analysis of Thymic Treg

Individual collagenase-treated, disaggregated thymi were stained for CD4 (GK1.1), CD8 and Foxp3 (FJK-16s) together with other markers including CD44 (IM78.1), CD62L (MEL14), CD69 (H1.2F3), CD103 (2E7), GITR (DTA-1), or Vβ panel of antibodies (BD Pharmingen).

### Histology

Formalin-fixed thymi from RelB^−/−^ mice were sectioned and stained with hematoxylin and eosin.

### Immunofluorescence and Confocal Microscopy

Tissues were harvested and immersed in OCT freezing medium and 10 µm sections prepared using a microtome. Sections were fixed in ice cold acetone and blocked with 5% BSA and 0.1% Tween-20, then were incubated with antibody in blocking buffer, washed and mounted with fluorescent mounting medium. They were visualized on an LSM-510-meta confocal microscope (Carl Zeiss Micro Imaging GmBH, Gottingen, Germany). Antibody clones include the following: MTS10, RelB (C-20), CD11c-APC (N418), CD4-PE (RM4-5), CD8-APC (53-6.7), Foxp3-Alexa-488 (FJK-16s), F480-Alexa-488 (BM8), CD11b-PE (Clone M1/70), and Gr1-APC (RB6-8C5). DAPI identified cell nuclei (13–15).

### Statistical Analysis

Unpaired, two-tailed, Student’s *t* tests, ∝ = 0.05, assessed whether the means of two normally distributed groups differed significantly. Mann–Whitney test (unpaired) was used for non-normally distributed means or for sample sizes <10. One-way ANOVA analysis with Bonferroni’s multiple comparison post-test compared multiple means. Significance is indicated as *p < 0.05, **p < 0.005, and ***p < 0.001. All error bars represent SEM.

## Results

### Preservation of the Medullary Niche for tTreg Development but Biased TCR Repertoire in RelB^−/−^ Mice Lacking AIRE^+^ mTECs

**Figure 1:**
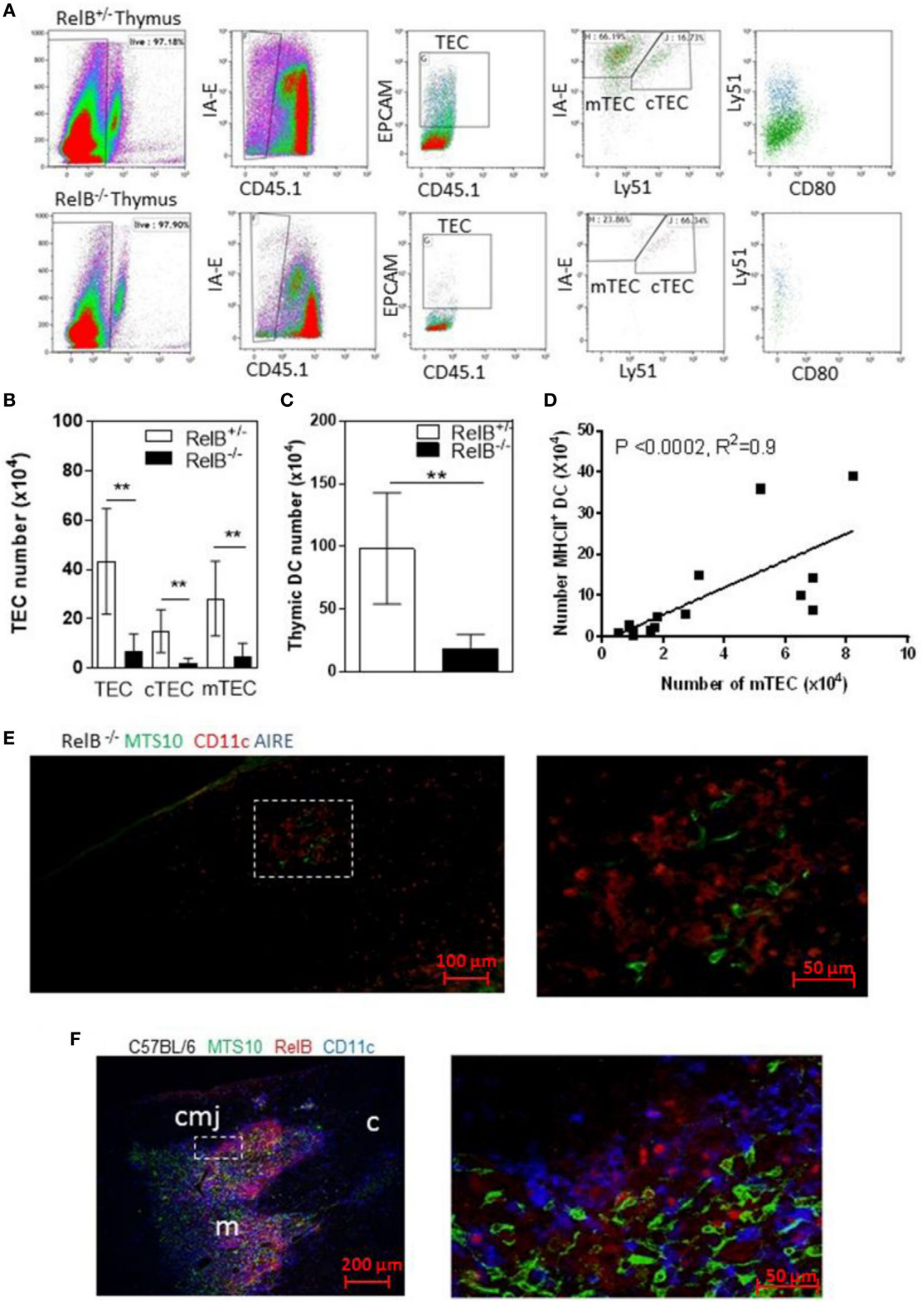
RelB deficiency reduces medullary thymic epithelial cell (mTEC) and dendritic cell (DC) numbers in thymus. **(A)** Reduced TECs, including cTECs [CD45^−^MHCII^+^ (IA-E) EPCAM^+^Ly51^+^] and mTECs (CD45^−^MHCII^+^EPCAM^+^Ly51^−^) in RelB^−/−^ thymi. **(B)** mTECs (MHCII^+^, Ly51^−^) and cTEC (MHCII^+^, Ly51^+^) and **(C)** MHCII^+^CD11c^+^ DC numbers in thymi from RelB^−/−^ and RelB^+/−^ mice; **p ≤ 0.01 (unpaired Student’s two-tailed *t* test). **(D)** Analysis of individual mice showing correlation of mTEC number and DC number; Spearman method. **(E)** Representative immunofluorescence staining of frozen thymic section from RelB^−/−^ mouse. MTS10^+^ mTECs (green) and CD11c^+^ DC (blue) are AIRE negative (red). **(F)** Representative immunofluorescence staining of frozen thymic section from C57BL/6 mice. Both CD11c^+^ DCs (blue) and MTS10^+^ mTECs (green) are RelB^+^ (red). RelB expression is highest in the corticomedullary junction (cmj). Medulla (m) and the cortex (c) are shown.

RelB is required for development of AIRE^+^ mTECs. RelB^−/−^ mice also have impaired negative selection, and T-cell-dependent multiorgan autoimmune disease (4, 16–18). RelB^−/−^ thymi had fewer total TECs including cTECs and mTECs, and fewer thymic DCs than in wild type (RelB^+/−^) mice (Figures 1A–C). The numbers of mTECs and DCs were correlated, with a Spearman coefficient of 0.9 (Figure 1D). As previously reported, RelB^−/−^ mice had reduced medullary size compared with RelB^+/−^ thymi, and AIRE staining was not detected in RelB^−/−^ medulla (Figures 1E,F; Figure S1 in Supplementary Material).

**Figure 2:**
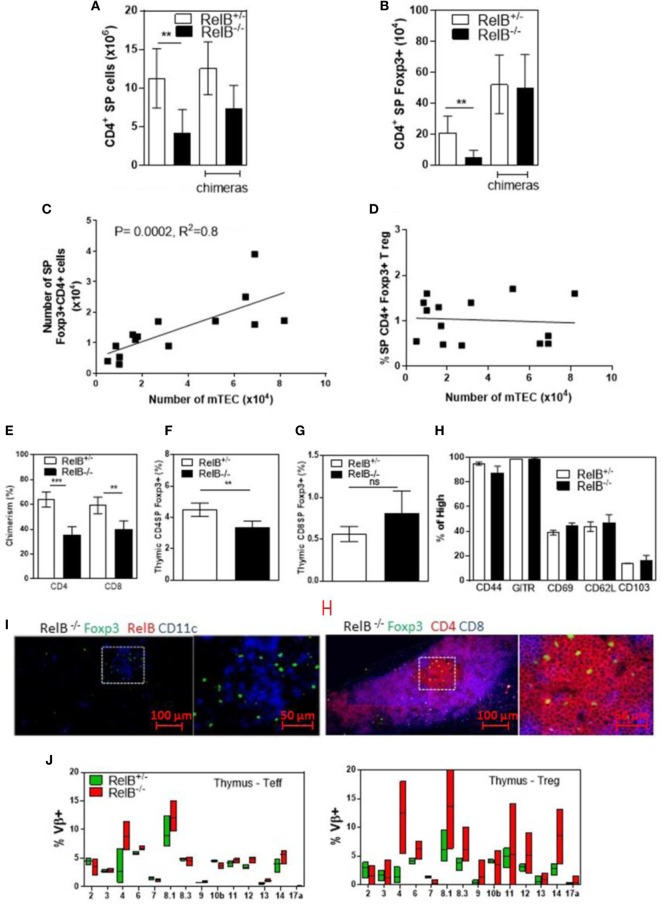
RelB deficiency reduces Treg numbers in thymus. SP CD4^+^ **(A)** and Foxp3^+^CD4^+^ **(B)** cell numbers in thymi from RelB^+/−^, RelB^−/−^, RelB^+/−^ bone marrow chimeras (BMC) and RelB^−/−^ BMC mice; **p ≤ 0.01 (unpaired Student’s two-tailed *t* test). **(C)** Analysis of individual mice showing correlation between medullary thymic epithelial cell (mTEC) number and Treg number; Spearman method. **(D)** Analysis of individual mice showing no change in Treg proportions relative to mTEC numbers. **(E)** Thymic Treg development in mixed RelB^−/−^/RelB^+/−^ (50:50) → RelB^+/−^ BMC: flow cytometry analysis of a representative BMC mouse showing proportions of RelB^+/−^ Treg (CD45.1) and RelB^−/−^ Treg (CD45.2) in SP CD4^+^ **(F)** and SP CD8^+^ **(G)** donor cells. **(H)** Percentage of thymic Treg from RelB^+/−^ and RelB^−/−^ mice expressing surface markers as determined by flow cytometry. (**I)** Thymic frozen sections from RelB^−/−^ mice showing CD11c^+^ DCs (blue) and Foxp3^+^ Treg (green) in the medulla and normal localization of SP CD4^+^ T cells (red). **(J)** Proportion of Vβ TCR in SP CD4^+^Foxp3^−^ and CD4^+^Foxp3^+^ T cells in thymus of RelB^+/−^ and RelB^−/−^ mice as determined by flow cytometry.

Consistent with the small size of the thymus, the numbers of CD4^+^ SP thymocytes and tTreg developing in RelB^−/−^ thymi were reduced (Figures 2A,B). mTEC numbers correlated with tTreg numbers but not proportion (Figures 2C,D). By contrast, the numbers of CD4^+^ SP thymocytes and tTreg isolated from thymi from RelB^−/−^ → RelB^+/−^ BMC and control RelB^+/−^ → RelB^+/−^ BMC were equivalent (Figures 2A,B). The BMC data indicate that RelB expression in non-mTECs, including DCs, did not influence Treg development. However, RelB^−/−^ tTreg had reduced competitive fitness compared with RelB^+/−^ Treg in 50:50 mixed BM chimera mice, indicating a minor cell-intrinsic role for RelB in tTreg development (Figures 2E–G; Figure S2 in Supplementary Material). Although fewer, tTreg developing in the absence of RelB expressed similar levels of CD44, GITR, CD69, CD62L, and CD103 to control mice (Figure 2H). SP and Foxp3^+^ thymocytes could be identified in the medulla of RelB^−/−^ mice (Figure 2I; Figure S3 in Supplementary Material). Cellular analysis of thymic tissue from RelB^+/−^ mice demonstrated a similar thymic medullary architectural organization with RelB expression and resident DCs within medullary regions (Figure 1F). RelB deficiency not only reduced tTreg development but also biased Vβ usage in Foxp3^+^ T cells in the thymus with expanded Vβ4, Vβ8.1, and Vβ14 TCR (Figure 2J). Together, these data indicate that although tTreg development is reduced when mTECs lack RelB, the medullary niche for tTreg development is preserved in RelB^−/−^ mice lacking AIRE^+^ mTECs and tTreg are phenotypically indistinguishable from RelB^+/−^ tTreg. However, they have a TCR bias consistent with expanded autoreactive clones (19–22).

### Expansion of Thymic DCs and of tTreg Are RelB Dependent

**Figure 3:**
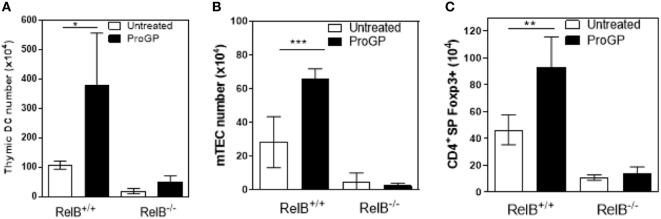
RelB expression is required for medullary thymic epithelial cell (mTEC)/dendritic cell (DC) expansion and increased Treg output in response to ProGP-1. Thymi from mice treated with ProGP-1 were harvested, collagenase digested, and analyzed by flow cytometry to determine the number of MHCII^+^CD11c^+^ DC **(A)**, mTEC **(B)**, and SP CD4^+^Foxp3^+^ Treg **(C)**; *p ≤ 0.05, **p ≤ 0.01, and ***p ≤ 0.001 (unpaired Student’s two-tailed *t* test).

The correlation between DCs, mTECs and tTreg and the chemokine downregulation in RelB^−/−^ thymus suggests that mTEC proliferation/survival might provide an appropriate environment to support DC seeding of the medulla and/or that expansion of DCs may be required to increase thymic mTEC and tTreg numbers and medullary size. We therefore tested whether the medulla could be expanded by inducing intra-thymic DC proliferation with Progenipoeitin-1 (ProGP-1: recombinant G-CSF and flt3-L) (23) administered to mice for 10 days. After ProGP-1, DCs expanded in RelB^+/+^ but not RelB^−/−^ thymus (Figure 3A). Consistent with our hypothesis, the number of mTECs and tTregs also increased only in RelB^+/+^ thymus (Figures 3B,C). There was no similar response to ProGP-1 in RelB^−/−^ thymus. Since DCs and tTreg numbers are not reduced in RelB^−/−^ → RelB^+/−^ relative to RelB^+/−^ → RelB^+/−^ chimeras these data indicate that mTEC RelB expression is required for mTEC/DC expansion and increased Treg output.

### Thymic Inflammation Associated With Atrophy in RelB^−/−^ Mice

**Figure 4:**
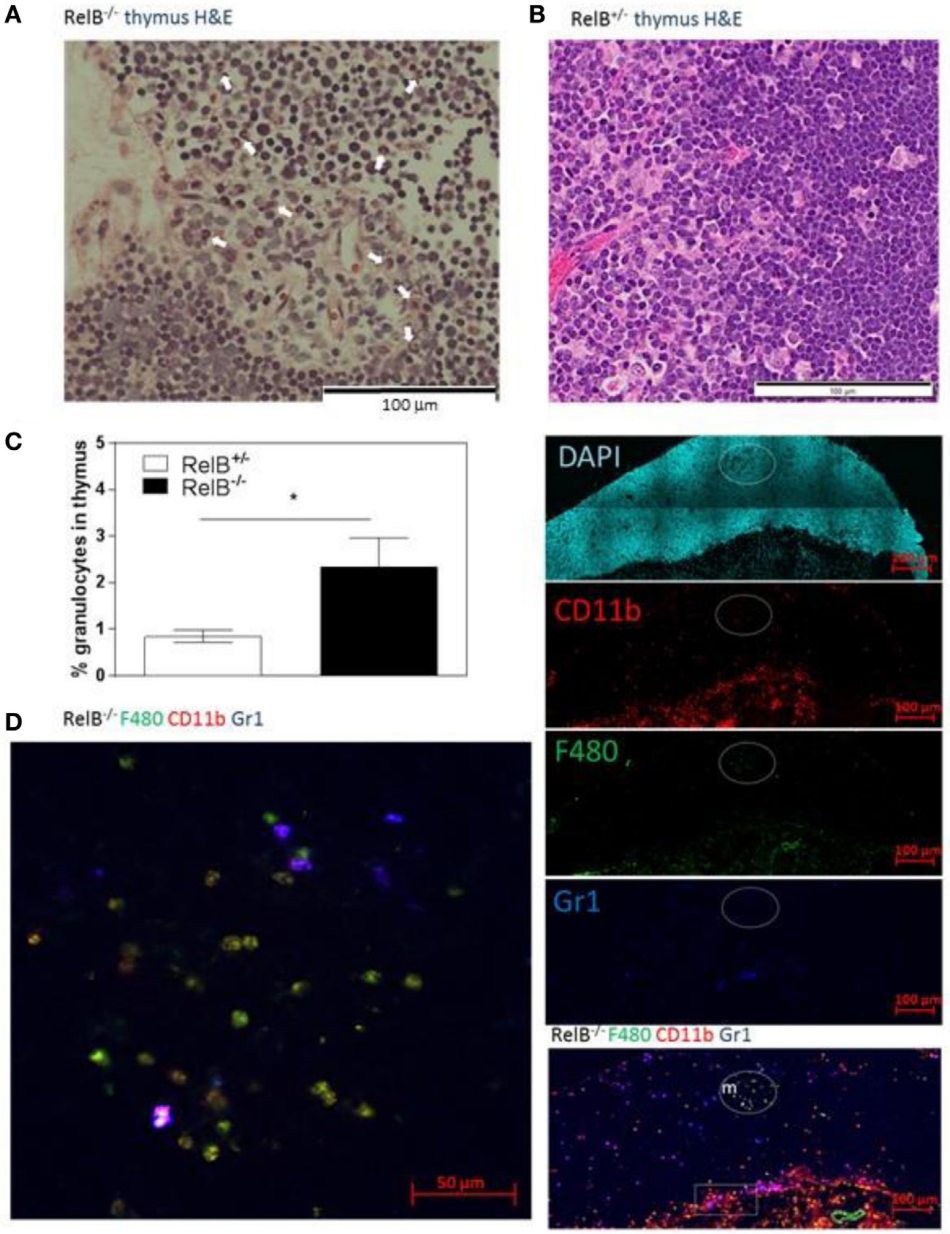
Thymic inflammation in RelB^−/−^ mice. Hematoxylin and eosin staining of RelB^−/−^ **(A)** and RelB^+/−^ **(B)** thymi. Arrows in panel **(A)** show granulocytes. **(C)** Thymi from RelB^−/−^ and RelB^+/−^ mice were collagenase-digested and disaggregated; cells were counted and stained for Ly6c/g, CD11b, and F480, then analyzed by flow cytometry; *p ≤ 0.05 (unpaired Student’s two-tailed *t* test). **(D)** Immunofluorescent staining of frozen thymic sections obtained from RelB^−/−^ mice for CD11b, F4/80, and Ly6c/g. CD11b^+^F480^+^ cells are seen in the medulla (m) and cortex, particularly adjacent to the capsule (sub-capsular inflammation marked by rectangle).

We noted that RelB^−/−^ thymi were not only small but also were infiltrated in the thymic capsular sinus by polymorphonuclear leukocytes, as identified by hematoxylin and eosin staining (Figure 4A), suggesting the possibility of inflammatory tissue damage. The percentage of CD11b^+^ granulocytes was increased in RelB^−/−^ thymi compared with RelB^+/−^ thymi (Figure 4B). The inflammatory CD11b^+^Ly6c/g^+^ granulocytic infiltrate accumulated around the cortex and adjacent to the capsule of RelB^−/−^ thymus but not RelB^+/+^ thymus (Figure 4C; Figure S4 in Supplementary Material). CD11b^+^ cells accumulating in the medulla were F480^+^, including Ly6c/g^−^ eosinophils and Ly6c/g^+^ neutrophils (Figure 4D). Thus thymi of RelB^−/−^ mice are affected by severe inflammatory cell infiltration.

### Granulocyte Depletion Preserves Thymic Function and Enhances tTreg Development in RelB^−/−^ Mice

**Figure 5:**
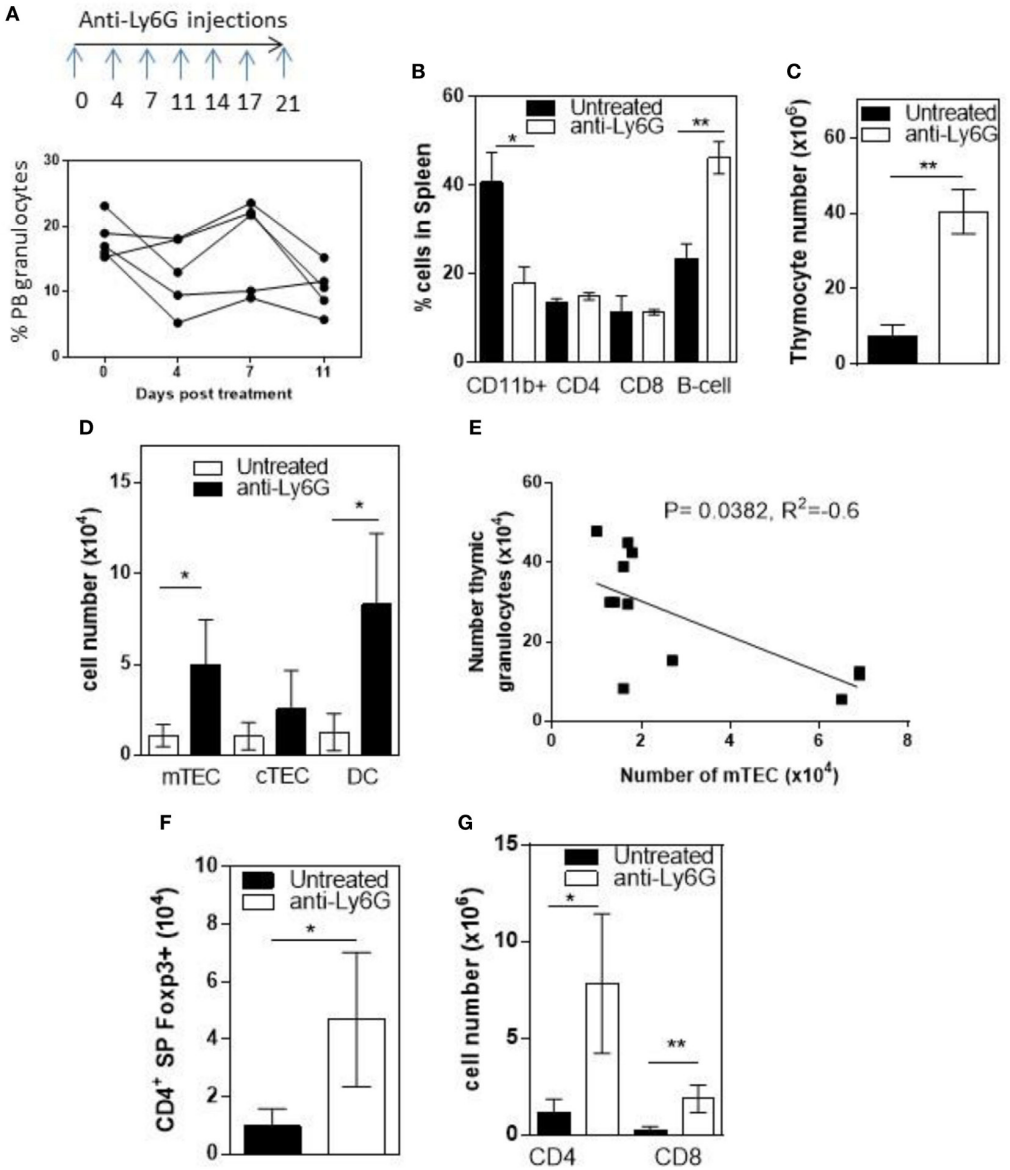
Depletion of neutrophils prevents thymic atrophy in RelB^−/−^ mice. RelB^−/−^ mice (6–8 weeks of age) were treated twice weekly for 4 weeks with depleting anti-Ly6G antibody. **(A)** Proportion of peripheral blood neutrophils (CD11b^+^, Ly6c^−^, and Ly6G^+^) as assessed by flow cytometry during depletion. **(B)** Proportion of immune cells in the spleen after 4 weeks of neutrophil depletion; *p ≤ 0.05 and **p ≤ 0.01 (unpaired Student’s two-tailed *t* test). Collagenase-digested thymi from anti-Ly6G-treated RelB^−/−^ mice or age-matched RelB^−/−^ mice were analyzed by flow cytometry to determine the number of thymocytes **(C)**, medullary thymic epithelial cells (mTECs), cTEC, MHCII^+^CD11c^+^ dendritic cell (DC) **(D)**, CD4 and CD8 SP cells **(G)**, and SP CD4^+^Foxp3^+^ Treg **(F)**; *p ≤ 0.05 and **p ≤ 0.01 (unpaired Student’s two-tailed *t* test). **(E)** Analysis of individual mice showing correlation of mTEC number with number of thymic granulocytes; Spearman method.

To determine whether infiltrating granulocytes suppressed thymic function, we treated 6- to 8-week-old RelB^−/−^ mice twice weekly for 4 weeks with anti-Ly6g mAb to deplete granulocytes. Flow cytometric analysis of peripheral blood demonstrated variable longitudinal reduction of Ly6g^+^ granulocytes (Figure 5A). In spleen, the proportion of CD11b^+^ granulocytes was reduced and the proportion of B cells increased after 4 weeks (Figure 5B; Figure S5 in Supplementary Material). Comparison of thymi from age-matched RelB^−/−^ mice with and without 4-week granulocyte depletion demonstrated an overall increase in thymocytes, mTECs, and thymic DCs, consistent with preservation of thymic medullary size in mice treated with anti-Ly6g mAb (Figures 5C,D). Furthermore, the number of granulocytes in the thymus correlated negatively with the number of mTECs in individual RelB^−/−^ mice (Figure 5E). The larger medullary niche afforded by the increased number of mTECs upon granulocyte depletion was associated with increased numbers of SP CD4^+^ and CD8^+^ T cells, including SP CD4^+^Foxp3^+^ tTreg (Figures 5F,G). These data demonstrate inflammatory, granulocyte-associated thymic atrophy in RelB^−/−^ mice, and that depletion of granulocytes attenuates medullary atrophy and improves thymic T cell output including tTreg. Together, these data indicate that granulocytes infiltrating the thymus of RelB^−/−^ mice accelerate thymic atrophy.

### Transfer of RelB^+^ DCs Preserves Thymic Structure due to Suppression of Autoimmune Inflammation in the Thymus

**Figure 6:**
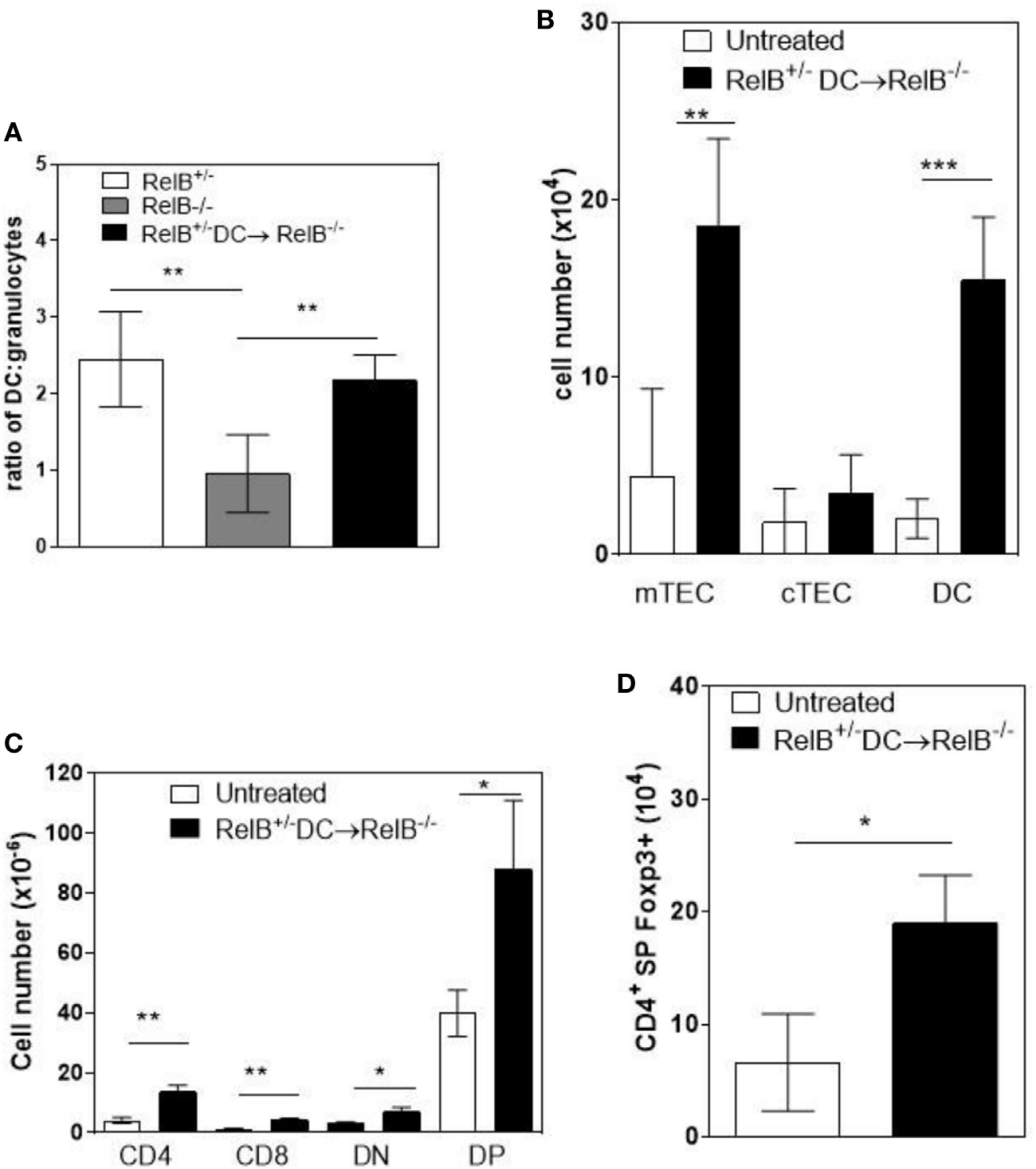
Transfer of RelB^+^ dendritic cells (DCs) preserves thymic structure. RelB^−/−^ mice were adoptively transferred with 5 × 10^6^ RelB^+/−^ DCs. **(A)** Thymi from RelB^+/−^ (8–10 weeks), untreated RelB^−/−^ (8–10 weeks), or RelB^+/−^ DC-treated RelB^−/−^ mice (8–10 weeks of age treated for 2 weeks) were harvested, collagenase digested, and analyzed by flow cytometry to determine the ratio of DCs (MHCII^+^CD11c^+^) to granulocytes (CD11b^+^CD11c^lo^); **p < 0.01 (one-way ANOVA followed by Bonferroni’s *post hoc* test). Thymi from untreated RelB^−/−^ (8–10 weeks) or DC-treated RelB^−/−^ mice (8–10 weeks of age treated for 2 weeks) were compared for numbers of medullary thymic epithelial cell (mTEC), cTEC, DC **(B)**, SP CD4, SP CD8, DN, DP **(C)**, and SP CD4^+^Foxp3^+^ Treg **(D)**; *p ≤ 0.05, **p ≤ 0.01, and ***p ≤ 0.001 (unpaired Student’s two-tailed *t* test).

A T cell-dependent, B cell-independent autoimmune inflammatory granulocytic infiltrate affects multiple organs in RelB^−/−^ mice (9, 24). Furthermore, adoptive transfer of low numbers of RelB-sufficient DCs to RelB^−/−^ mice induced dominant Treg-mediated suppression of autoimmune disease, as measured by weight gain, improved clinical scores, and reduced granulocyte infiltration of the spleen (9). Therefore, we examined whether thymic medullary atrophy in RelB^−/−^ mice could be prevented by adoptive transfer of DCs, through dominant tolerance of autoimmune disease. In RelB^−/−^ mice treated with RelB^+/−^ DCs, the numbers of infiltrating CD11b^+^Gr1^+^ myeloid cells decreased in the thymus, relative to DCs (Figure 6A). The absolute numbers of mTECs and DCs in the medulla increased with DC adoptive therapy (Figure 6B), and the development of SP T cells, including tTreg, increased (Figures 6C,D). These data strongly suggest that the thymic inflammation and accelerated atrophy in RelB^−/−^ mice is autoimmune. Furthermore, thymic DCs, mTECs, and tTreg output can be preserved by RelB^+/−^ DC immunotherapy, which controls granulocytic inflammation in RelB^−/−^ mice. Thus, the medullary niche for mTEC, DC, and tTreg survival and expansion can be preserved in RelB^−/−^ thymus either by depletion of granulocytes or suppression of autoimmune disease using DC immunotherapy.

## Discussion

Medullary TECs and DCs support the development of tTreg in the thymus. Several studies have analyzed the role of mTECs in tTreg development in RelB-deficient thymus because of the severe reduction in mTECs. However, the severe thymic medullary atrophy and multiorgan autoimmune disease in RelB^−/−^ mice (5) limits interpretation of effects of RelB on tTreg development. Unexpectedly, considering the reduction in proportion and numbers of tTreg, thymi isolated from RelB^−/−^ mice were appropriately organized, with medullary structures containing DCs, SP CD4^+^ cells and Foxp3^+^ tTreg. RelB^−/−^ mTECs were AIRE^−^. RelB^−/−^ tTreg were phenotypically similar to RelB^+/−^ tTreg. However, relative to control mice, the numbers of tTreg were reduced in RelB^−/−^ mice and unchanged in RelB^−/−^ BMC. Granulocytes infiltrated the RelB^−/−^ thymic cortex, capsule, and the medulla, associated with an enrichment in genes mediating myeloid cell chemotaxis. Importantly, RelB^−/−^ mice had profound granulocyte-induced inflammatory thymic medullary atrophy, which could be treated by granulocyte depletion or RelB^+^ DC immunotherapy, with concomitant recovery of tTreg numbers. While the exact mechanism by which the infiltrating granulocytes promote this thymic atrophy is unknown, RelB is known to repress *Il1b* and granulocyte extracellular traps could act as a platform for IL-1 activation (25, 26). These data demonstrate that RelB deficiency profoundly affects tTreg development due to reduced size of the medullary niche, as a result of inflammatory thymic medullary atrophy. This conclusion has important implications for autoimmune diseases where polymorphisms in genes such as *Traf6, Relb*, and *Rel* reduce expression and transcriptional activity of RelB in thymus (27, 28), including loss of function. Rare RelB mutations have indeed been identified in humans, with similar reduction in thymic size, disorganized corticomedullary architecture, altered thymic T cell maturation, reduced T cell output, and skewed T cell repertoire (29).

We show here that accelerated autoimmunity was a further consequence of this medullary atrophy, including lymphopenia due to reduced output of CD4^+^ SP cells and decreased TCR diversity skewed toward Vβ4, very likely due to limited peripheral tissue antigen expression by mTECs within the niche, and lack of AIRE expression (30–33). In consequence, self-reactive peripheral Teff and Treg expansion and organ-specific inflammatory disease were evident (9). With respect to the role of thymic DCs in tTreg development, the normal output of tTreg in RelB^−/−^ BMC demonstrates that if mTECs and the medullary niche are intact then RelB expression by DCs is not required for normal tTreg development. Even the failure to differentiate mature mTECs in the absence of RelB itself had a minor impact on tTreg development, since tTreg output increased with two strategies to reduce granulocytic infiltration of the thymus. Rather, the autoimmune consequences of the defect in negative selection, the functional deficiency of pTreg and granulocytic inflammation, together, reduced tTreg output. This interpretation is consistent with experimental data demonstrating that tTreg development is impaired in RelB^−/−^ thymic grafts into wt mice, where the medulla was atrophied (8). Furthermore, in a more direct experiment, mice with conditional deletion of *Traf6* (upstream of RelB) in mTECs had a similar reduction in mTEC differentiation, reduction in AIRE^+^ mTECs, and reduced medullary size without thymic inflammation. This reduction in medullary size was associated with a 50% reduction in tTreg output. Organ-specific autoimmune disease in these mice developed much later than in RelB^−/−^ mice, as peripheral tolerance and pTreg function was shown to be normal.

AIRE increases expression of peripheral antigens by mTECs, including insulin, salivary protein-1 and fatty acid binding protein. AIRE also promotes apoptosis of mTECs, facilitating cross presentation of antigen to DCs (34). AIRE^−/−^ mice differ from RelB^−/−^ mice, in that they retain mature mTECs and they can express non-AIRE encoded peripheral tissue antigens, such as C-reactive protein and GAD67. AIRE^−/−^ mice also have reduced medullary accumulation of thymic DCs that, together with mTECs, promote tTreg development (10, 11). AIRE^−/−^ mTECs still attract DCs to medullary regions independently of AIRE and AIRE-induced chemokines, such as XCL1, CCL19, CCL21, and CCL25 (11), some of which were decreased in RelB^−/−^ mice. However, medullary DC accumulation was reduced when mTECs were reduced. The reduced thymic medullary area for negative selection and Treg development, and the reduction in mTEC AIRE are together likely to explain the organ-specific autoimmune disease in RelB^−/−^ mice. The increased severity of autoimmune disease in RelB^−/−^ relative to AIRE^−/−^ mice is also consistent with the additional Treg dysfunction in RelB^−/−^ mice. In AIRE^−/−^ mice pTreg function is normal and TCR sequencing of Treg and Teff clones from organs of TCR-mini transgenic mice crossed with AIRE^−/−^ mice showed that Treg TCR diversity is maintained independent of AIRE, with little overlap in the TCR repertoire of Teff and Treg clones isolated from disease-affected salivary glands (35).

ProGP-1 and FLT3L have been shown to expand peripheral CD11c^+^CD8^+^MHCII^hi^ DCs in mice (23). We show that proGP-1 also expands thymic DCs, with concomitant increases in mTECs and tTreg. Thymic renewal driven by proGP-1 was dependent on RelB, potentially due to a requirement for RelB expression for development of bipotent TEC progenitors (17). While FLT3L administration expanded peripheral DCs and pTreg numbers, this was not accompanied by increases in thymic Treg (36). Furthermore, G-CSF had no impact on thymocyte progenitor activity in rats (37). Our data therefore implicate G-CSF in the expansion of mTEC progenitors, but only when associated with FLT3L-mediated DC expansion, and suggest the hypothesis that expansion of the appropriate DC subset in the thymus can signal proliferation of mTECs from progenitors (38), providing a niche for tTreg development.

In summary, genetically mediated central tolerance defects may be accelerated by thymic inflammation where polymorphisms impair mTEC development. Given that thymic inflammation may be triggered by infection, this has important implications for the development of childhood autoimmune diseases associated with neutrophilia and eosinophilia (39). Furthermore, accelerated thymic atrophy may be reversible at least in part by therapies that enhance pTreg function and/or that limit innate inflammatory cell-mediated thymic inflammation.

## Ethics Statement

This study was carried out in accordance with the recommendations of the UQ Animal Ethics committee. The protocol was approved by the UQ Animal Ethics committee.

## Author Contributions

Concept and design of study: BOS, AC, and RT. Data collection, analysis, and interpretation: BOS, RR, SY, MM, AM, AC, and RT. Manuscript preparation: BOS and RT.

## Conflict of Interest Statement

The authors declare that the research was conducted in the absence of any commercial or financial relationships that could be construed as a potential conflict of interest.
