# Supplementary material for: Autoimmune-Mediated Thymic Atrophy Is Accelerated but Reversible in RelB-Deficient Mice
Source: Front Immunol. 2018 May 22;9:1092. doi: 10.3389/fimmu.2018.01092 (PMC5972300; doi:10.3389/fimmu.2018.01092)

Supplementary Figure 1. RelB expression in thymus of C57BL/6 mice. Representative immunofluorescence staining of frozen thymic section with MTS10 (mTECs - green), CD11c+ (DC - blue) and AIRE (red). AIRE expression is observed in mTEC but not CD11c+ DC.

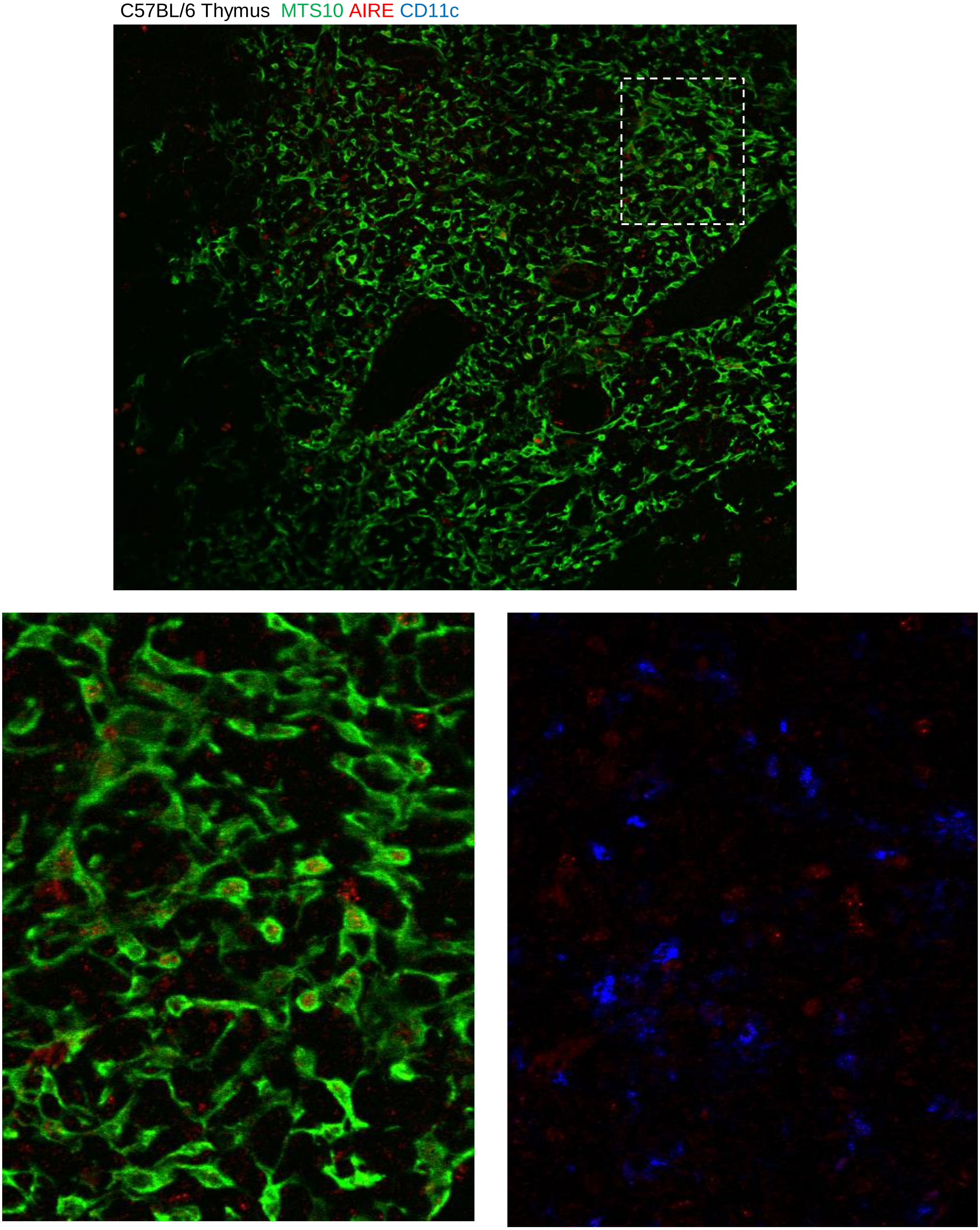

Supplementary Figure 2. Thymic Treg development in mixed RelB-/-/RelB+/- (50:50) → RelB+/- BMC. Flow cytometry analysis of a representative BMC mouse showing proportions RelB+/-Treg (CD45.1) and RelB-/- Treg (CD45.2) in SP CD4+ and SP CD8+ donor cells.

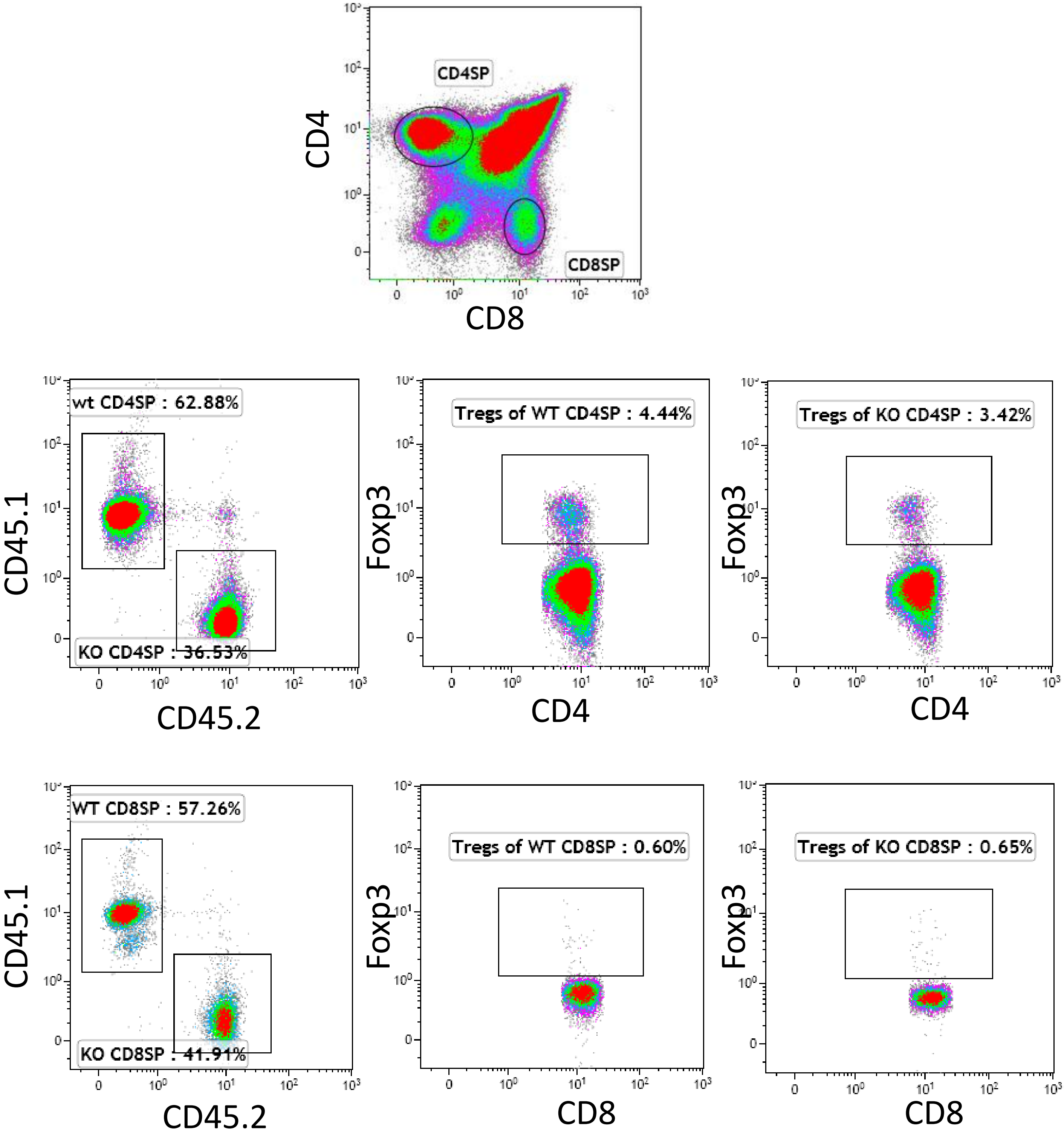

Supplementary Figure 3. FoxP3 expression in thymus of C57BL/6 mice. Representative immunofluorescence staining of frozen thymic section with FoxP3 (green), CD11c+ (DC - blue) and RelB (red) (upper panel) and FoxP3 (green), CD4 (red) and CD8 (blue) (lower panel).

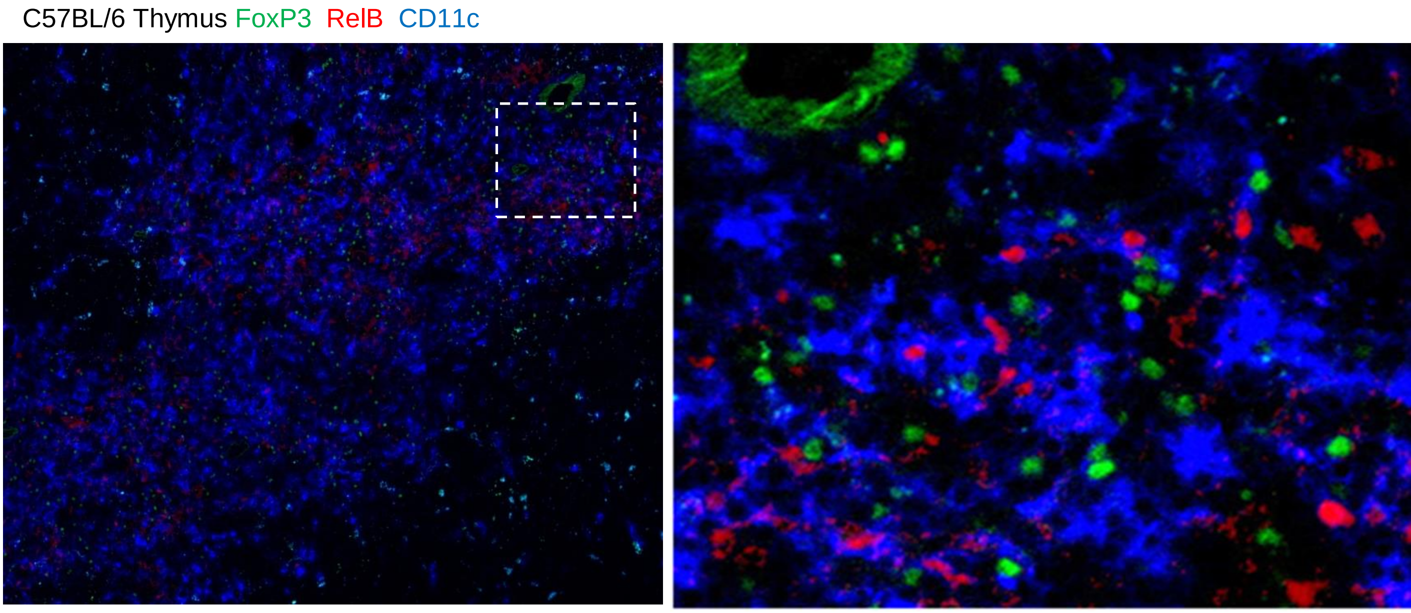

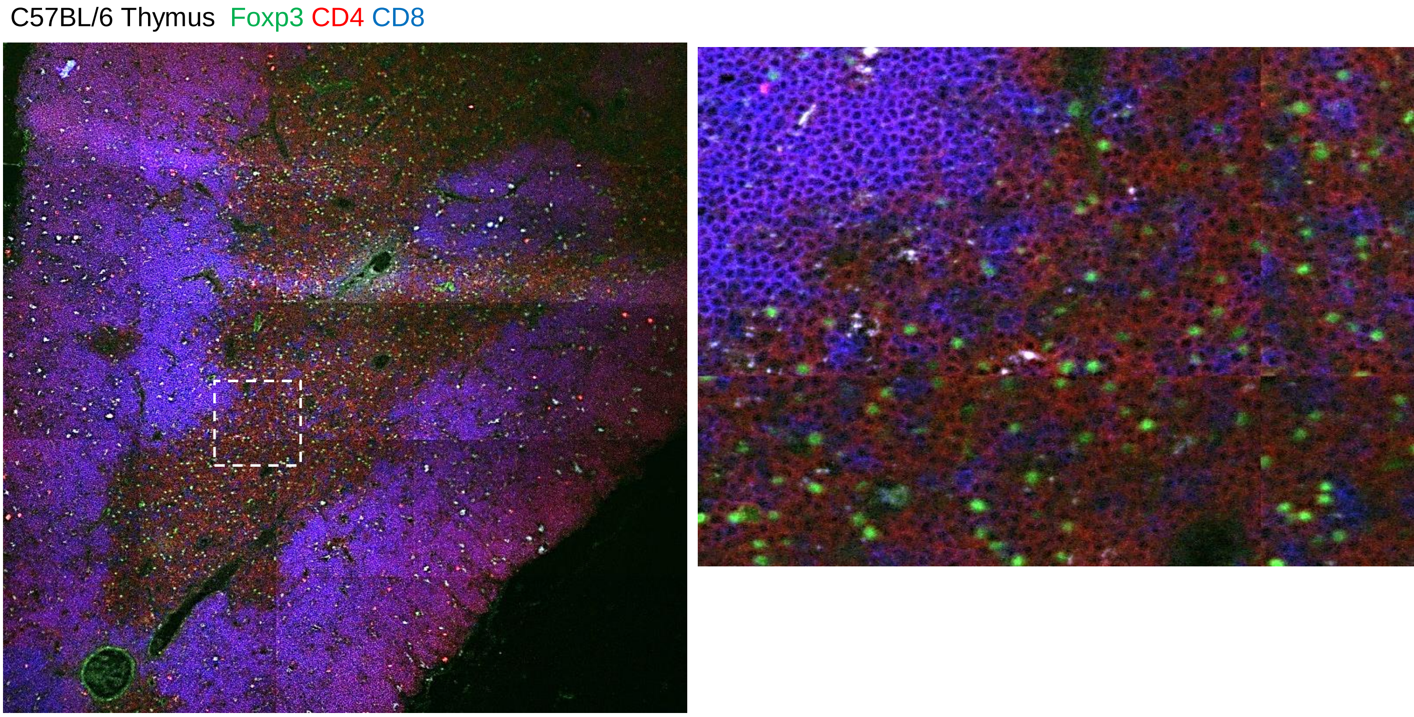

Supplementary Figure 4. Thymic granulocytes in C57BL/6 mice. Representative immunofluorescence staining of frozen thymic section with DAPI (cyan), CD11b (red) and F480 (green) Gr1 (blue).

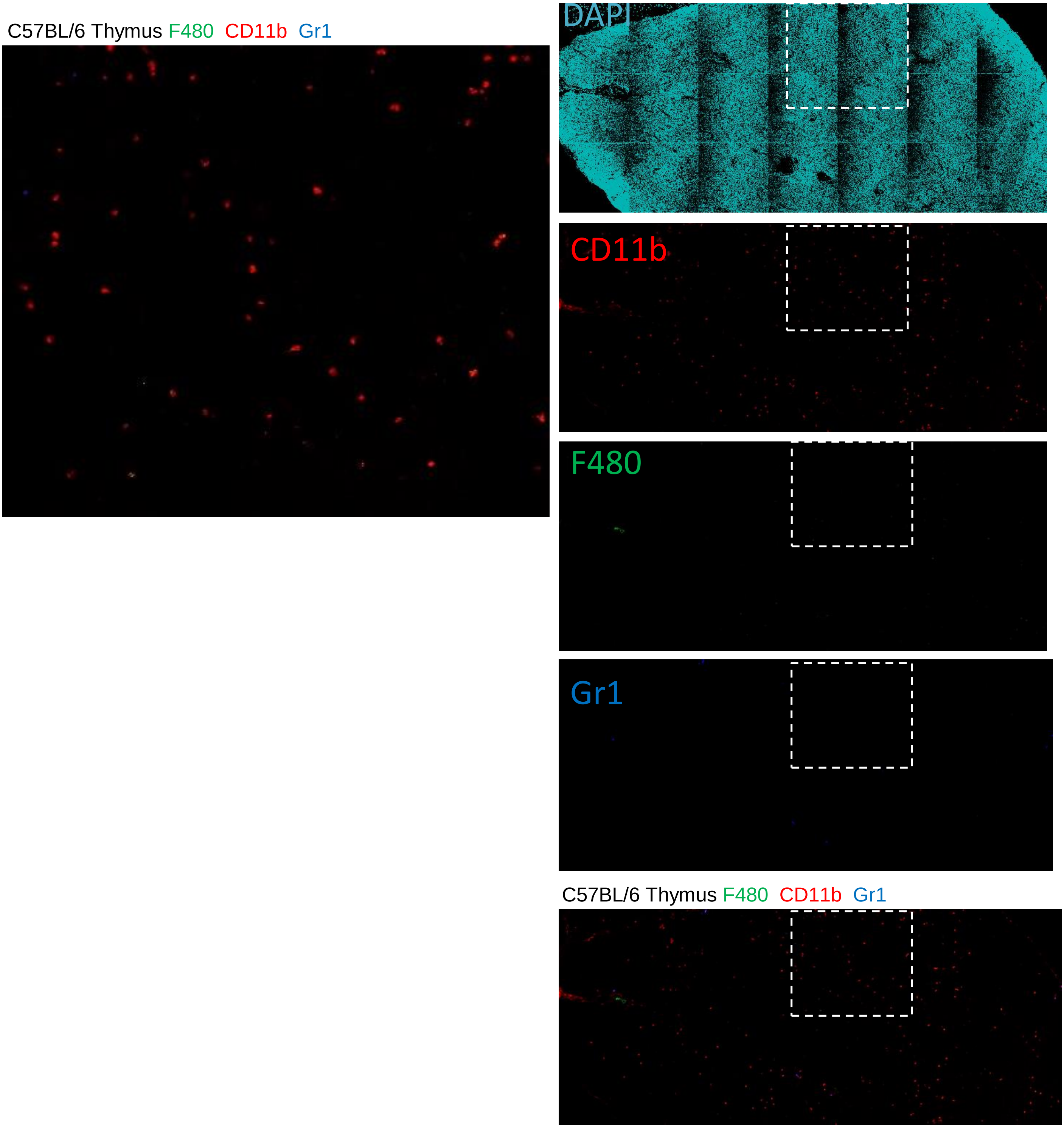

Supplementary Figure 5. Flow cytometric gating of splenocytes from neutrophil depleted RelB-/-mice. RelB-/-mice depleted of neutrophils with anti-Ly6G antibody, isotype antibody or untreated for 4weeks, sacrificed and splenocytes analysed by cytometry. Shown are live cells gated on CD11b+ cells and stained for Ly6G and Ly6C. Neutrophils are CD11b+Ly6G+Ly6G-.

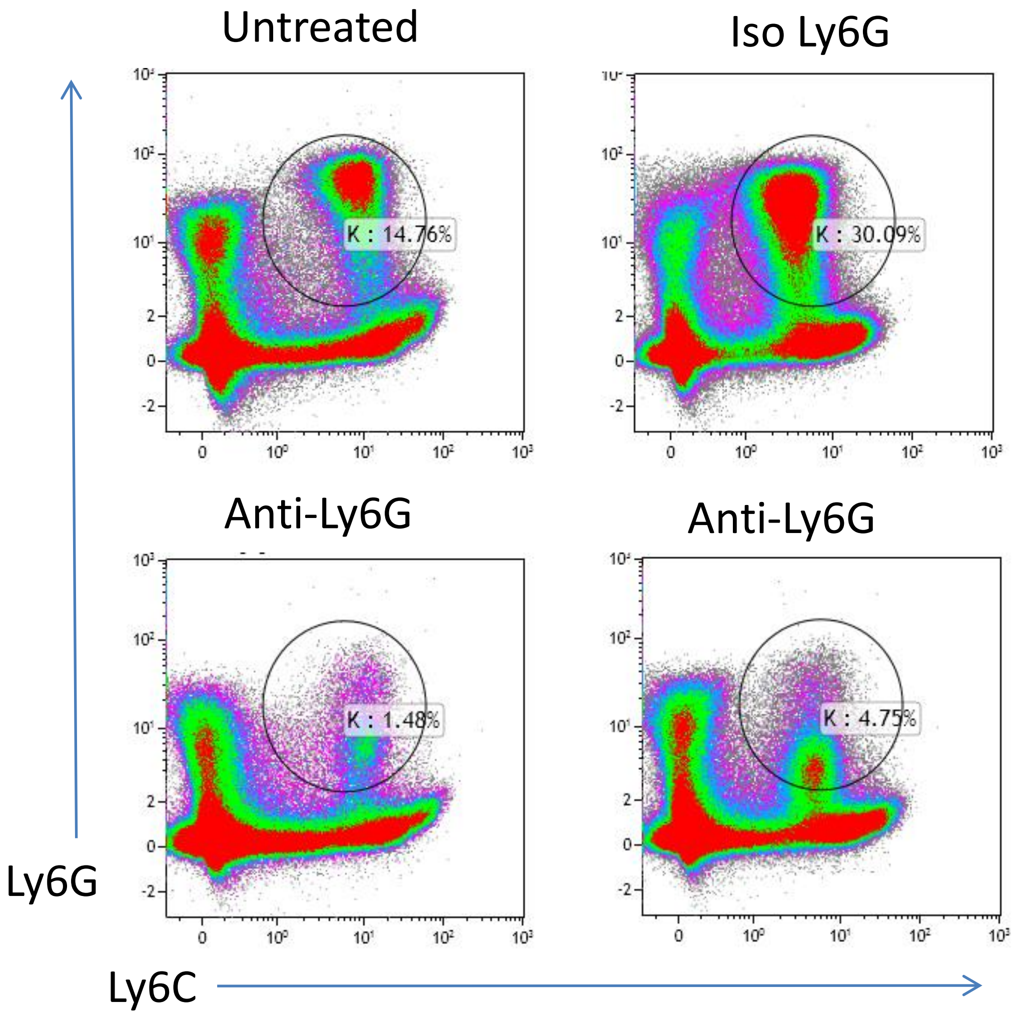

Supplement: Supplementary file 1 [file data_sheet_1.PDF]
